# Lactate induces aberration in the miR-30a–DBF4 axis to promote the development of gastric cancer and weakens the sensitivity to 5-Fu

**DOI:** 10.1186/s12935-021-02291-2

**Published:** 2021-11-10

**Authors:** Tengkai Wang, Rui Ji, Guanqun Liu, Beilei Ma, Zehua Wang, Qian Wang

**Affiliations:** 1 Department of Internal Medicine, Qilu Hospital, Shandong University, 107 West Wenhua Road, Jinan, 250012 Shandong P.R. China; 2 Department of Gastroenterology, Qilu Hospital, Shandong University, Jinan, Shandong P.R. China; 3 Department of Clinical Laboratory, Qilu Hospital, Shandong University (Qingdao), 758 Hefei Road, Qingdao, Shandong P. R. China; 4 Department of Clinical Laboratory, Qilu Hospital, Shandong University, 107 Wenhuaxi Road, Jinan, 250012 Shandong P.R. China

**Keywords:** Gastric cancer, DBF4, miR-30a, Lactate, Proliferation, migration,5-Fu

## Abstract

**Background:**

Gastric cancer (GC) is one of the most common malignancies, molecular mechanism of which is still not clear. Aberrant expression of tumor-associated genes is the major cause of tumorigenesis. DBF4 is an important factor in cancers, although there is yet no report on its function and molecular mechanism in GC.

**Methods:**

The expression of DBF4 in tumor tissues or cells of GC was detected by qRT-PCR and western blotting. Gastric cancer cell line MGC-803 and AGS were transfected with DBF4 siRNA or overexpression vector to detect the function of DBF4 in proliferation, migration and the sensitivity to 5-Fu with CCK-8 assay, colony formation assay, transwell assay, and wound healing assay. miR-30a was found to be the regulator of DBF4 by online bioinformatics software and confirmed with qRT-PCR, western blot and dual-luciferase reporter assays.

**Results:**

In our study, increased expression of DBF4 in GC tissues was first identified through The Cancer Genome Atlas (TCGA) and later confirmed using specimens from GC patients. Furthermore, functional experiments were applied to demonstrate that DBF4 promotes cell proliferation and migration in GC cell lines, moreover weakens the sensitivity of MGC803 and AGS cells to 5-Fu. We further demonstrated that miR-30a showed significantly lower expression in GC cells and inhibited the expression of DBF4 through 3ʹ-UTR suppression. Furthermore, rescue experiments revealed that the miR-30a-DBF4 axis regulated the GC cell proliferation, migration and the sensitivity to 5-Fu. The important composition in tumor microenvironment, lactate, may be the primary factor that suppressed miR-30a to strengthen the expression of DBF4.

**Conclusions:**

Taken together, our study was the first to identify DBF4 as a regulator of cell proliferation and migration in GC. Furthermore, our study identified the lactate-miR-30a-DBF4 axis as a crucial regulator of tumor progression and the tumor sensitivity to 5-Fu, which maybe serve useful for the development of novel therapeutic targets.

## Introduction

Gastric cancer (GC) is the fifth most common malignancy and ranks as the third leading cause of cancer-related death worldwide, with over 1 million estimated new cases each year [1–3]. Although there have been recent advances in GC diagnosis and therapy, the 5-year survival rate remains below 30 % for GC patients [4]. Therefore, it necessary to determine the underlying pathogenic molecular mechanisms of GC to develop effective and novel strategies for treatment [5, 6].

A hallmark of cancer cells is aberrations in DNA replication, a major cause of genomic instability and tumorigenesis [7]. DNA replication is initiated by assembly of the checkpoint effector enzyme cell division cycle 7-related protein kinase (CDC7), which then phosphorylates the minichromosome maintenance (MCM) helicase complex on the MCM2 and MCM4 subunits [8–10]. The main regulatory activation subunit of CDC7 is DBF4, which is a highly conserved protein that represents an essential checkpoint at the G1/S transition [11]. DBF4 is reported to be scarcely expressed in normal tissues but significantly overexpressed in many cancer cells [10, 12]. Additionally, upregulation of DBF4 is associated with lower relapse-free survival in many cancers, indicating that enhanced DBF4 may be a signal of malignancy in humans [13]. However, the effect of DBF4 in GC and the underlying molecular mechanisms regulating DBF4 remains largely unclear, and must be further elucidated.

MicroRNAs (miRNAs) are a class of endogenous non-coding small RNAs containing 18–25 nucleotides. The miRNAs regulate the expression of target genes by directly binding to the 3ʹ untranslated region (3ʹ-UTR) of mRNA, leading to degradation and/or inhibition of translation [14, 15]. Previous studies have identified many tumor suppressor genes as targets of microRNAs, and aberrant expression of miRNA has been reported in the development and progression of many types of cancer. Thus, microRNA dysregulation has the potential to serve as a novel biomarker for disease [15–20]. miR-30a was reported to be responsible for the intestinal transcripts in stomach cells during the progression of intestinal metaplasia [21]. A previous study reported that miR-30a-5p may also play a suppressive role in GC [22]. However, there is poor characterization of the existing data related to the regulatory role of miR-30a-5p in GC growth and the regulation of miR-30a-5p expression.

Therefore, we conducted the first study on DBF4 in GC with the aim of elucidating the functional significance and molecular mechanisms of miR-30a-5p expression.

## Materials and methods

### Patient samples

We collected GC tissues and corresponding adjacent noncancerous tissues from Qilu Hospital, Shandong University (Shandong, China). The samples were collected from GC patients, immediately frozen, and stored at −80 °C until use. All samples were collected for research use only. The experimental protocols of this study were approved by the ethics committees of Qilu Hospital, Shandong University.

### Cell culture

Human GC cell lines (MGC-803 and AGS) were purchased from the Cell Resource Center (Shanghai, China). MGC-803 cells were cultured in Dulbecco’s Modified Eagle Medium (DMEM; Gibco, USA) and AGS cells were cultured in Ham’s F-12 K Medium with 10 % fetal bovine serum (FBS; Biological Industries, USA) and 100 U/mL penicillin-streptomycin (Solarbio, China). Cells were maintained in an incubator with 5 % CO_2_ at 37 °C.

### Cell transfection

Small interfering RNA (siRNA) targeting DBF4 (si-DBF4), negative control siRNA (si-NC), miR-30a mimics with corresponding negative controls (NC), the PEX-DBF4 overexpression vector, and the PEX vector were purchased from Genepharma (Shanghai, China). The mutated vector was constructed by KeyGEN BioTECH (Jiangsu, China). All of the constructs were verified by sequencing. Lipofectamine 2000 reagent (Invitrogen, Carlsbad, CA, USA) was used to perform cell transfection, in accordance with the manufacturer’s protocol.

### Quantitative real-time polymerase chain reaction (qRT-PCR)

TRIzol reagent (Invitrogen) was applied to harvest total RNA from GC cells and GC tissues. The All-in-One™ miRNA qRT-PCR Detection Kit (GeneCopoeia, FulenGen, China) or Prime Script™ RT reagent kit (Takara, Shiga, Japan) was used to generate cDNA. The qRT-PCR was performed on the ABI Real-Time PCR System. The primers used were synthesized by Sangon (Shanghai, China) as follows: DBF4 (forward, 5′-TGCAGTCCATTTGATGTAGACAAG-3′; reverse, 5′- GAGGTTCCACCATACTTATCGCC-3′), β-actin (forward, 5′- AGTTGCGTTACACCCTTTC-3′; reverse, 5′- CCTTCACCGTTCCAGTTT-3′). The conditions used for qRT-PCR were as follows: 94 °C for 10 s followed by 40 cycles of 95 °C for 10 s and 60 °C for 20 s. The relative expression of DBF4 and miR-30a was calculated using the 2^−ΔΔCt^ method and β-actin or U6 snRNA served as an internal control.

### Western blotting

The proteins from tissues and GC cells were extracted using cell lysis buffer (Beyotime, Shanghai, China) and rationed by BCA Reagent kit (Beyotime, Shanghai, China). Protein (50 µg) was loaded into 10 % sodium dodecyl sulfate-polyacrylamide gel electrophoresis (SDS-PAGE) gel and transferred onto polyvinylidene fluoride (PVDF) membranes (Millipore, Burlington, MA, USA). The PVDF membranes were blocked using Tris-buffered saline with Tween 20 (TBST) with 5 % nonfat milk at room temperature for 2 h. Membranes were then washed with phosphate-buffered saline with Tween 20 (PBST) and incubated overnight at 4 °C with the corresponding primary antibodies: anti-DBF4 (1:2,000; ABclonal, Wuhan, China), anti-cyclin-A (1:2,000; ABclonal, China), anti-PCNA (1:2,000; ABclonal, China), and anti-β-actin (1:5,000; ABclonal, China). Membranes were washed and then incubated with matched secondary antibodies conjugated with horseradish peroxidase (1:5,000; ABclonal, China) for 1 h at room temperature. Membranes were visualized using an enhanced chemiluminescence (ECL) kit (Thermo Fisher Scientific, USA). β-actin was used as a loading control.

### Dual-luciferase reporter assay

The interaction between miR-30a and DBF4 was predicted by the bioinformatics database. MGC-803 and AGS cells were seeded in 24-well plates. GC cell were co-transfected with reporter plasmid and miR-30a mimics or negative control using Lipofectamine 2000. After 48 h, cells were lysed with passive lysis buffer for 10 min at 4 °C. Luciferase activity was assessed using the Dual Luciferase Assay Kit (Promega, Madison, WI, USA), according to the manufacturer’s protocol. Firefly luciferase was normalized to Renilla luciferase activity.

### CCK-8 assay

The treated cells were seeded in 96-well plates and were incubated with 100 µL DMEM, containing CCK-8 solution (10 µL/well) for 30 min at 37 °C. The absorbance was measured at 450 nm with a spectrophotometer (Molecular Devices, San Jose, CA, USA). All experiments were repeated three times in triplicate.

### Transwell assay

Transwell chambers with 8-µm pore filters (BD Biosciences, San Jose, CA, USA) were used to determine the migration of transfected GC cells. Transfected GC cells at a density of 5 × 10^4^cells/chamber were added to the upper chamber of each transwell containing 1 % FBS medium. Medium with 10 % FBS was added to the bottom chamber as a chemoattractant. The cells were nurtured at 37 °C in a 5 % CO_2_ humidified atmosphere. The migration of transfected GC cells onto the lower surface of the membrane were visualized by staining with 0.1 % Crystal Violet for 30 min. The number of migrating transfected GC cells was counted using an inverted microscope (Olympus, Tokyo, Japan).

### Woud healing assay

The transfected cells were re-digested and seeded into a 24-well cell plate. After the cells adhere to the wall, a 10 µl sterile pipette tip was used to draw a straight line in the cell well. Rinse the floating cells repeatedly with sterile PBS and take pictures under the microscope. Later, change the medium to DMEM medium containing 1 % FBS and incubate for 24 h under 5 % CO2 37 °C. After 24 h, rinse repeatedly with sterile PBS, and take pictures under the microscope after removing the non-adherent cells. Cell migration was expressed as the migration rate: (original scratch area-new scratch area)/original scratch area ×100 %.

### Cell proliferation with 5-Fu treatment

Cell proliferation was evaluated using a Cell Counting Kit-8 assay. According to the manufacturer’s instructions, cells were plated in 96-well plates at an initial number of 5 × 10^3^ cells per well. Then, 10µL of CCK-8 solution was added to each well followed by incubation at 37℃ for 2 h. Sample absorbances were measured at 450nm using a microplate reader. Then CCK-8 solution was thrown away and cells were treated with different concentrations of 5-Fu for 48 h. After that absorbances of each well were measured using the same way. Taken cells without 5-Fu treatment as a control group, the relative cell viability was calculated according to the formula: relative cell viability (%) = OD experiment/OD control × 100 %.

### Statistical analysis

Comparisons between different groups were analyzed with GraphPad Prism v8.0 software (GraphPad Software, La Jolla, CA, USA) using unpaired *t*-tests. Data are presented as means ± standard deviation. Linear regression analysis was used to analyze the correlation between DBF4 and miR-30a expression in GC samples. Statistical significance was reported as highly significant using ^*^P<0.05, ^**^P< 0.01, ^***^P< 0.001, or ^****^P< 0.0001.

## Results

### DBF4 is upregulated in human gastric cancer

**Fig. 1:**
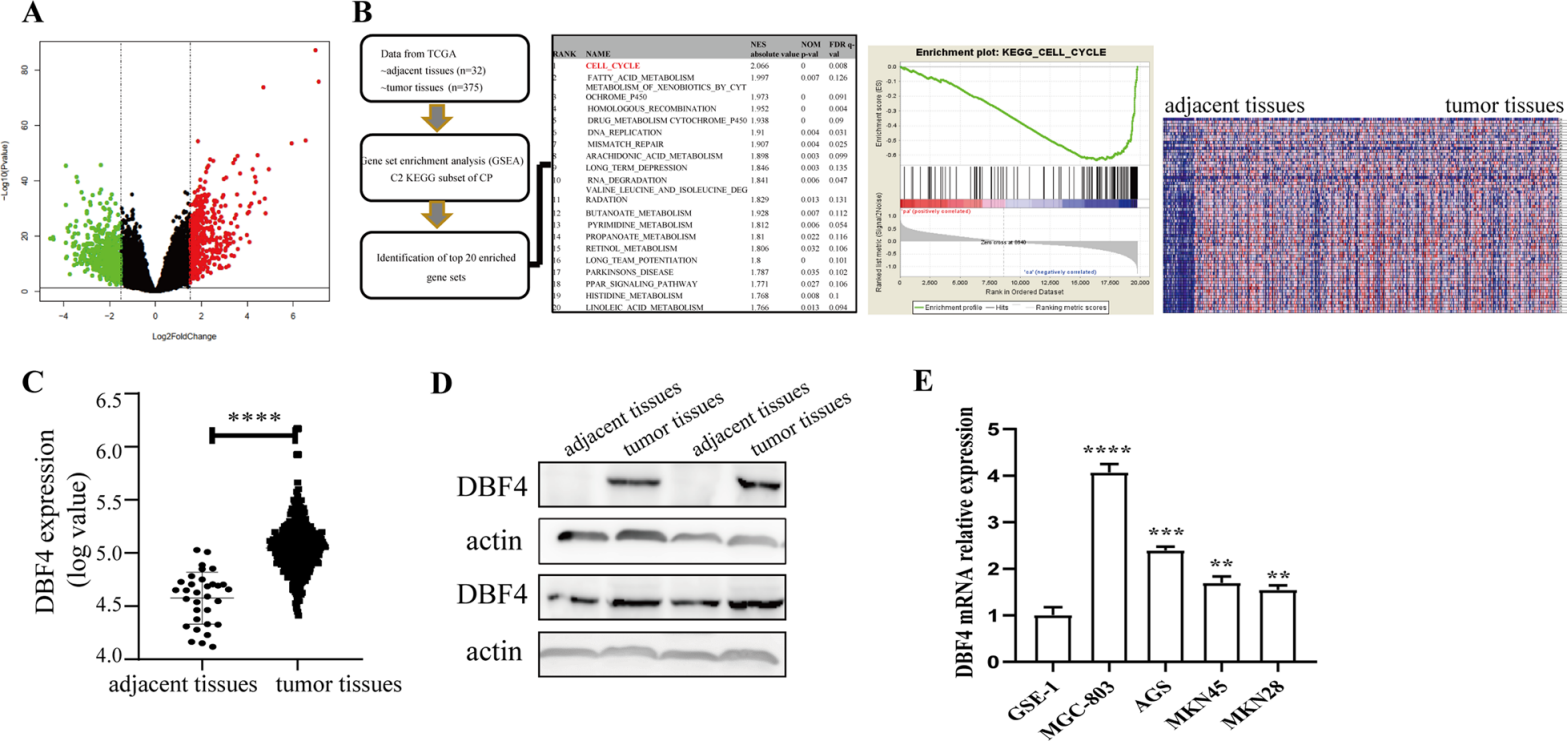
DBF4 is upregulated in GC.** A** The volcano map shows the expression variations of genes in GC tissues compared to matched adjacent normal tissues from TCGA. **B** Gene set enrichment analysis (GSEA) of differentially expressed genes [Enrichment Score (ES) = 0.634, Normalized Enrichment Score (NES) = 2.066, Nominal *p*-value = 0.0, FDR *q*-value = 0.008] (left) and the heat map shows differentially expressed genes in the KEGG_CELL_CYCLE (right). **C** Analysis of DBF4 mRNA expression in 375 GC tissues and 32 adjacent tissues. **D** DBF4 protein expression was detected by western blotting in four paired GC samples. **E** DBF4 expression in normal cell line GES-1 and GC cell lines MGC-803, AGS, MKN45 and MKN28 was measured by qRT-PCR. Data are displayed as the mean ± SD of three independent experiments. ^**^P < 0.01, ^***^P< 0.001, ^****^P< 0.0001

To explore gene expression profiles in GC, we performed gene set enrichment analysis (GSEA) with data from The Cancer Genome Atlas (TCGA; Fig. 1A, B). The analysis showed that the CELL_CYCLE signatures were significantly enriched in gastric tumor cells compared with adjacent tissues cells. We chose one of the most differentially expressed genes, DBF4, as our candidate for further experiments. DBF4 is a principle regulator of DNA replication, but its significant has not been previously explored in GC. Statistical analysis of DBF4 expression in 32 adjacent tissues and 375 tumor tissues from TCGA showed that DBF4 was dramatically upregulated in human GC (Fig. 1C). To further confirm the results from the TCGA analysis, an additional cohort with four pairs of GC tissues and adjacent normal tissues were collected to analyze DBF4 expression by western blots. As shown in Fig. 1D, DBF4 was increased significantly in GC tumor tissues, in accordance with the results from TCGA database. qRT-PCR was then employed to evaluate DBF4 expression in GC cells and normal cells, finding that DBF4 was potently up-regulated in GC cell lines (Fig. 1E). Collectively, these findings evinced that DBF4 was highly expressed in GC.

### DBF4 promotes the proliferation of gastric cancer cells

**Fig. 2:**
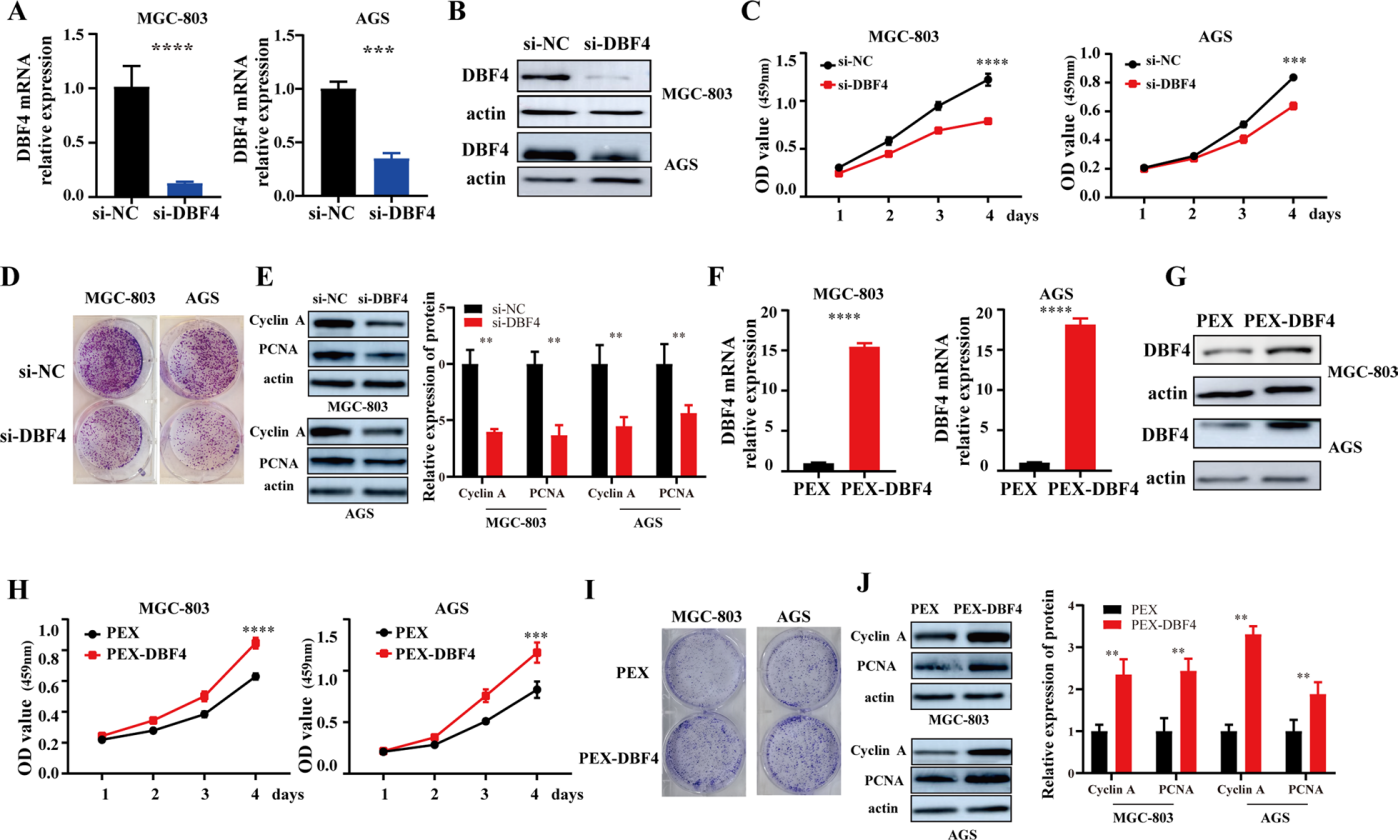
DBF4 promotes the proliferation of GC cells. **A**, **F** The qRT-PCR was conducted to measure DBF4 expression in MGC-803 (left) and AGS (right) cells transfected with si-DBF4 (**A**) or DBF4 overexpression vector (**F**). **B**,** G** DBF4 protein levels were detected by western blots in MGC-803 (above) and AGS (below) cells transfected with si-DBF4 (**B**) or DBF4 overexpression vector (**G**). **C**,** H** Cell growth analysis by CCK-8 assay in MGC-803 and AGS cells after DBF4 silencing (**C**) or DBF4 overexpression (**H**). **D**,** I** Colony formation assays were used to measure the proliferation of MGC-803 (left) and AGS (right) cells transfected with si-DBF4 (**D**) or DBF4 overexpression vector (**I**). Western blotting assays were used to analyze the expression of cyclin A and PCNA in MGC-803 and AGS cells transfected with si-DBF4 (**E**) or DBF4 overexpression vector (**J**). Data are displayed as the mean ± SD of three independent experiments. ^***^P< 0.001, ^****^P< 0.0001

To investigate the biological role of DBF4 in GC, we designed siRNAs to specifically target DBF4 and transfected the siRNAs into MGC-803 and AGS cells. The qRT-PCR and western blot analyses showed that the expression of DBF4 was significantly decreased in DBF4-siRNA-transfected cells (Fig. 2A, B). Next, we used CCK-8 assays and colony formation assays to detect proliferation of GC cells with decreased expression of DBF4. These assays showed that GC cell proliferation was decreased in the si-DBF4 group compared with the control group (*P* < 0.01; Fig. 2C, D). Expression of PCNA and cyclin-A was also decreased in GC cells transfected with si-DBF4 (Fig. 2E). To determine whether DBF4 overexpression had the opposite effect, we constructed the DBF4 overexpression vector, PEX-DBF4, and transfected this vector or the control vector (PEX) into GC cell lines (MGC-803 and AGS). The transfection efficiency was validated by qRT-PCR and western blotting (Fig. 2F, G). Functionally, the CCK-8 assay and colony formation assay suggested that overexpression of DBF4 enhanced GC cell proliferation (Fig. 2H, I). Expression of PCNA and cyclin-A was increased in GC cells with DBF4 overexpression, as detected by western blotting (Fig. 2J). Together, these data demonstrated that DBF4 enhanced GC cell proliferation.

### Expression of DBF4 weakens the sensitivity of MGC803 and AGS cells to 5-Fu

**Fig. 3:**
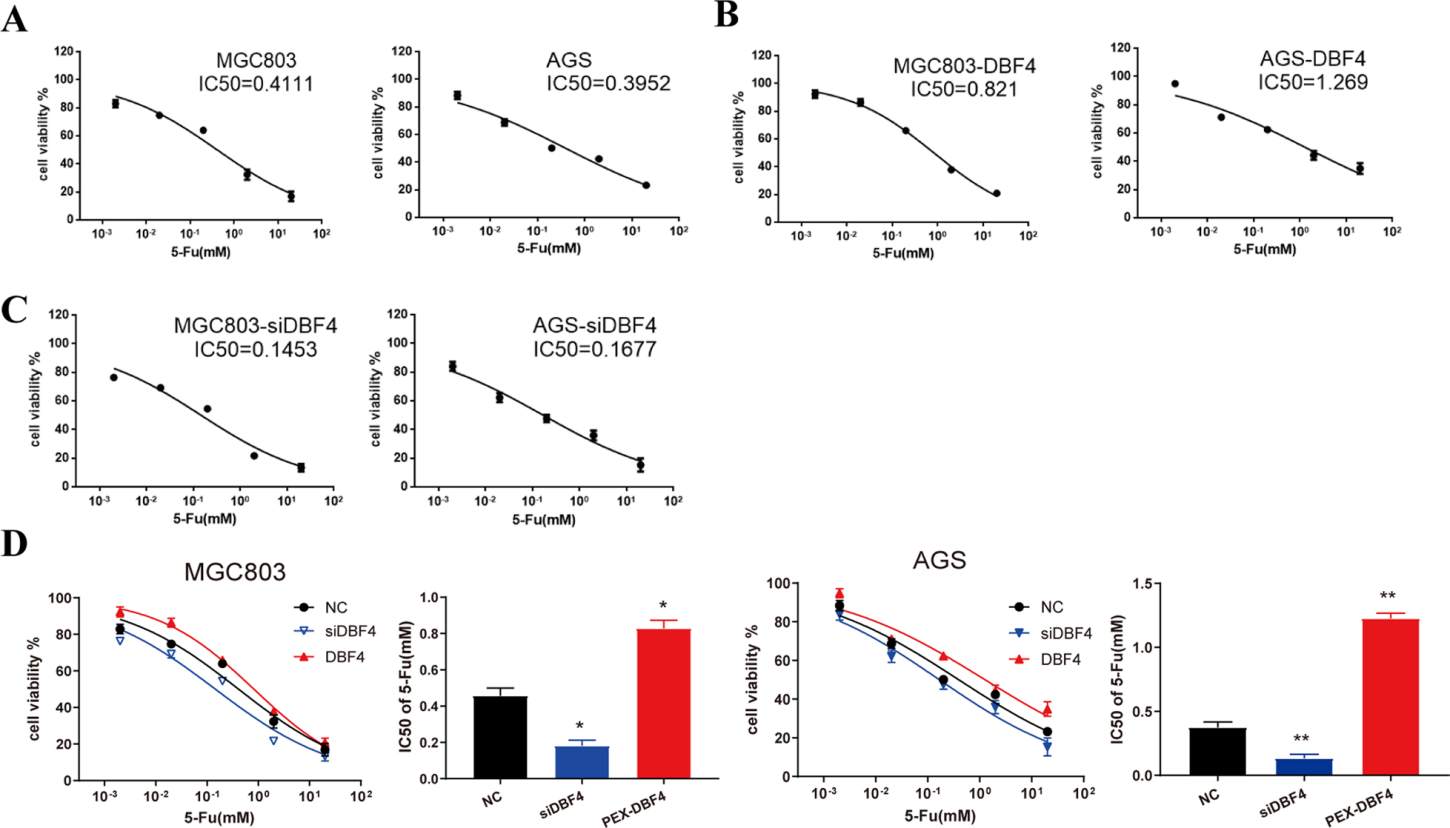
DBF4 weakens the sensitivity of MGC803 and AGS cells to 5-Fu. MGC803 and AGS cells were transfected with plasmid or siRNA for DBF4 (DBF4 or siDBF4) for 24 h. MGC803, AGS cells (**A**), DBF4-overexpressing MGC803-DBF4, AGS-DBF4 cells (**B**) and MGC803-siDBF4, AGS-siDBF4 cells (**C**) were incubated with control medium or various concentrations of 5-Fu as indicated at 37℃ for 48 h. After incubation, cell viability was measured by a CCK-8 assay. **D** Analysis for the IC50 of 5-Fu in treated cells. Results are expressed as percent of control and represent the mean±SD

5-fluorouracil (5‐Fu) is considered as one of the first‐line chemotherapy drugs in advanced gastric cancer(GC). First, the effect of 5‐Fu treatment on cellular proliferation‐associated MGC803 and AGS GC cells was assessed by CCK‐8 kit. As shown in Fig. 3 A, MGC803 and AGS cells were exposed to a series of concentrations of 5‐Fu for 48 h, taking the cell viability of the control sample (without 5‐Fu) as 100 %, viability of MGC803 and AGS cells was inhibited significantly after 5‐Fu treatment with the half‐maximal inhibitory concentration (IC50) values of 0.4111 and 0.3952 mM respectively. Then, DBF4 overexpression was performed in vitro based on MGC803 and AGS cells by plasmid transfection and IC 50 values of DBF4‐overexpressing MGC803-DBF4 and AGS-DBF4 cells were detected by the same way with 0.821 and 1.269 mM separately (Fig. 3B). Meanwhile, short interference siRNA for DBF4(siDBF4) were applied to knockdown the expression of DBF4 inMGC803 and AGS cells. IC50 values of MGC803-siDBF4 and AGS-siDBF4 cells were detected by the same way with 0.1453 and 0.1677 mM separately (Fig. 3C). Taken together, analysis for the IC 50 of 5‐Fu in treated cells (Fig. 3D) demonstrated that as an oncogene, DBF4 weakened the sensitivity of MGC803 and AGS GC cells to 5-Fu.

### DBF4 promotes the migration of gastric cancer cells

**Fig. 4:**
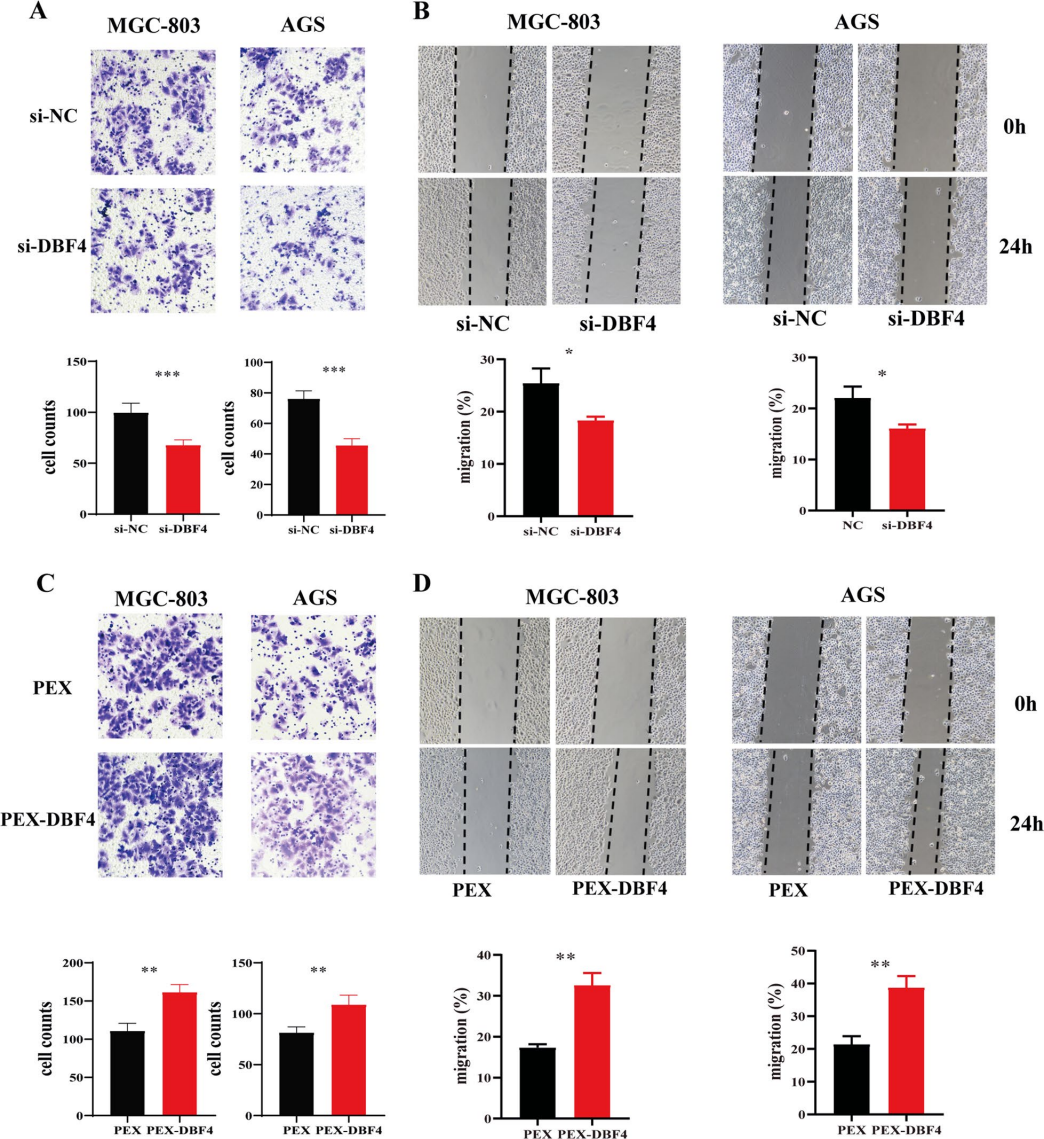
DBF4 enhanced the migration of GC cells. **A**,** B** MGC-803 and AGS cells were transfected with si-NC and si-DBF4. Transwell assay (**A**) and would healing assay (**B**) were applied to detect the migration of gastric cancer cells. **C**, **D** MGC-803 and AGS cells were treated with DBF4 overexpression. The migratory ability of gastric cancer cells were determined by transwell assay (**C**) and wound healing assay (**D**). Data are displayed as the mean ± SD of three independent experiments. ^**^P< 0.01, ^***^P< 0.001

To investigate the biological function of DBF4 in GC, transwell assays and woud healing assay were used to determine the migration of MGC-803 and AGS cells. As shown in Fig. 4A, B, silencing of DBF4 repressed the migration of both gastric cancer cells. To further confirm the results, DBF4 was overexpressed in MGC-803 and AGS cells and the migration of both GC were detected by transwell assays and woud healing assay. Consistent with the results above, the migratory ability of MGC-803 and AGS cells was strongly enhanced following DBF4 overexpression (Fig. 4C, D). Collectively, these experiments indicated that DBF4 also perform an important role in migration of GC cells.

### The miR-30a inhibits the expression of DBF4

**Fig. 5:**
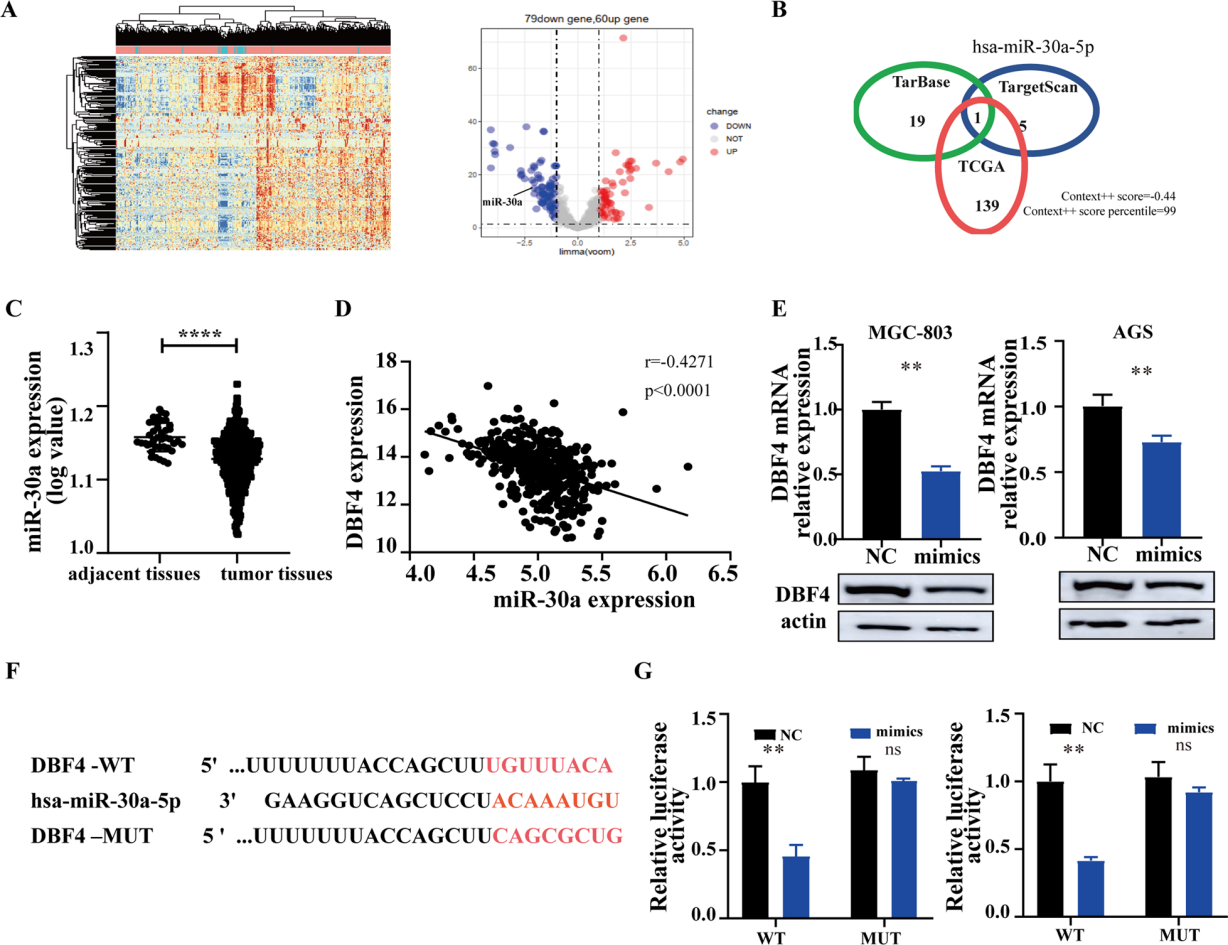
The miR-30a inhibits the expression of DBF4. **A** The heat map (left) and volcano map (right) show significantly downregulated and upregulated miRNAs in GC tissues from TCGA. **B** A Venn diagram shows the overlap of target miRNAs of DBF4, predicted by TargetScan, Tarbase, and the miRNA prolife from TCGA. **C** The expression levels of miR-30a in GC tissues from TCGA were analyzed. **D** The correlation between DBF4 and miR-30a expression in GC tissues was assessed by Pearson’s correlation method. **E** The expression of DBF4 in MGC-803 and AGS cells transfected with miR-30a or NC was detected by qRT-PCR and western blotting. **F** Schematic illustration of the binding site between miR-30a and wild-type (WT) or mutant (Mut) DBF4 3ʹ-UTR. **G** Relative luciferase activity of WT or Mut DBF4 3ʹ-UTR in MGC-803 and AGS cell lines after overexpression of miR-30a. Data are presented as the mean ± SD (n = 3). ^**^P < 0.01, ^****^P< 0.0001

The miRNAs are involved in the regulation of various biological processes such as cell proliferation [19], they regulate expression of many oncogenes and tumor suppressor genes, and they have been reported to play key roles in various human cancers, including GC [16, 17]. We first analyzed the miRNA expression profile in 436 GC tissues and 41 adjacent tissues tissues from TCGA. A total of 1881 miRNAs were expressed in these tissues and 139 showed differential expression. Of these, 79 miRNAs were downregulated and 60 were upregulated in GC (fold change ≥ 2 or ≤ 0.5, *P* < 0.05; Fig. 5A). A heat map was created showing differentially expressed microRNAs in GC tissues, relative to matched normal tissues (Fig. 5A). To determine the target regulatory miRNA for DBF4, two databases were screened, including TargetScan (https://www.targetscan.org) and TarBase (http://starbase.sysu.edu.cn/starbase2/). The only overlapping result from the two databases was miR-30a-5p (Fig. 5B). As shown in Figs. 4B and 5A, miR-30a-5p also shows significant differential expression in GC. Statistical analysis showed that miR-30a expression was significantly decreased in GC tumor tissue (Fig. 5C). In addition, the expression level of DBF4 was negatively correlated with the expression level of miR-30a in GC tissues, based on the data from TCGA (Fig. 5D). To further confirm the effect of miR-30a on DBF4 expression, both MGC-803 and AGS cells were transfected with miR-30a mimics and negative controls. DBF4 expression was analyzed by qRT-PCR and western blotting assays, which showed that DBF4 expression was significantly downregulated in GC cells transfected with miR-30a mimics. This suggested that DBF4 may be the target of miR-30a in GC (Fig. 5E). Moreover, bioinformatics analysis predicted a putative 8-mer-binding site with miR-30a in the 3ʹ-UTR of the DBF4 transcript (Fig. 5F). Dual-luciferase reporter assays were performed to determine whether miR-30a directly targets DBF4. Overexpression of miR-30a significantly decreased the luciferase activity of wild-type DBF4 in GC cells, but had no effect on luciferase activity of mutant DBF4 in GC cells (Fig. 5G), demonstrating that miR-30a specifically binds to the 3ʹ UTR of miR-30a. Taken together, these data suggested that miR-30a, one of the most differentially downregulated miRNAs in GC, directly regulated DBF4 expression.

### DBF4 overexpression restored the effects of miR-30a on gastric cancer cells

**Fig. 6:**
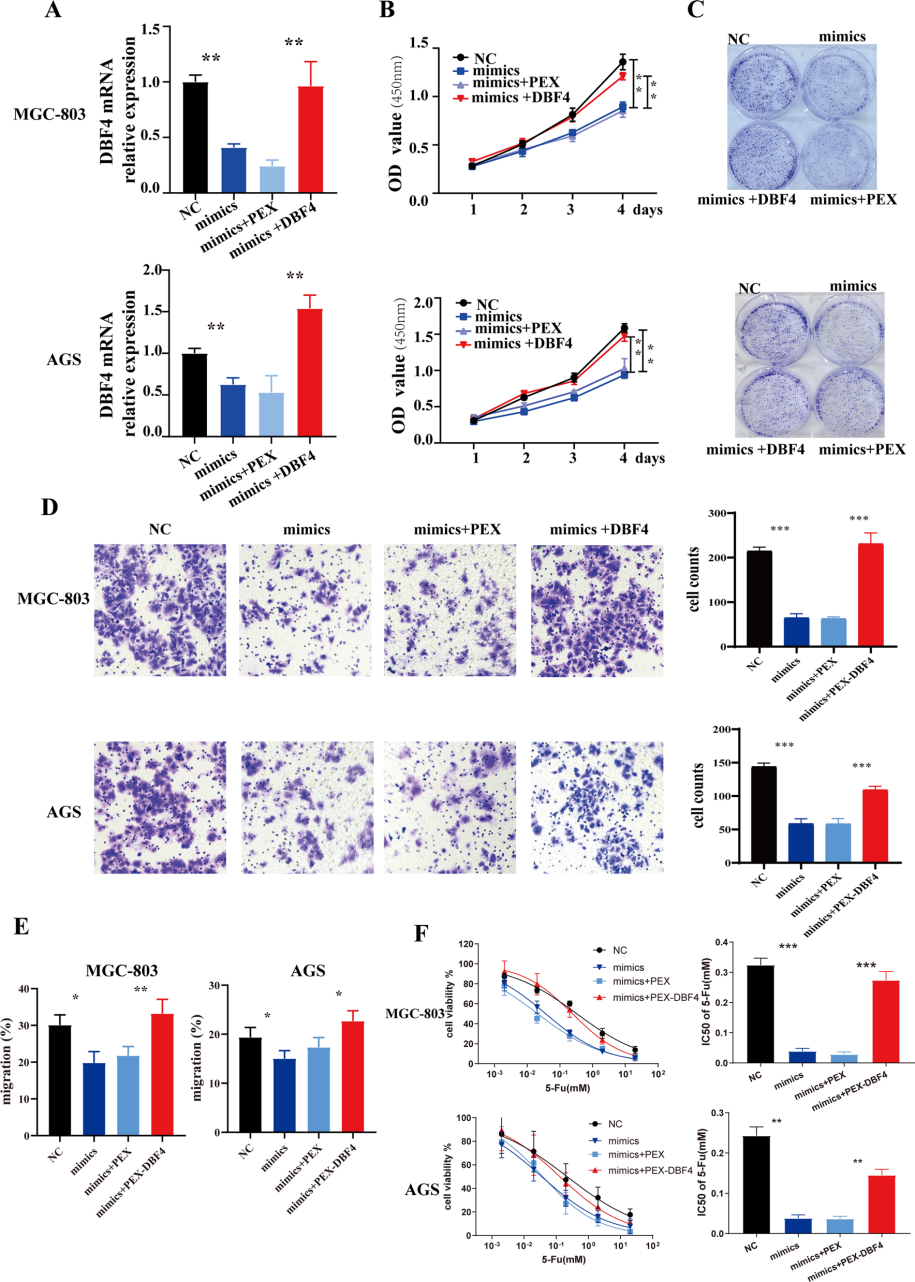
Overexpression of DBF4 restores the effects of miR-30a upregulation on GC cells. MGC-803 and AGS cells were transfected with miR-NC, miR-30a, miR-30a+PEX, or miR-30a+PEX-DBF4. **A** qRT-PCR analysis was used to determine DBF4 mRNA expression levels in MGC-803 (above) and AGS (below) cells. **B**,** C** The proliferation of MGC-803 and AGS cells was assessed using CCK-8 (**B**) and colony formation (**C**) assays. Transwell assay (**D**) and wound healing assay (**E**) were performed to detect the migration of gastric cancer cells. **F** The IC 50 of 5-Fu in the two GC cells introduced with miR-NC, miR-30a, miR-30a+PEX, or miR-30a+PEX-DBF4. Data are presented as the mean ± SD (n = 3). ^**^P < 0.01

Having confirmed the interaction of miR-30a and DBF4, next we performed rescue experiments to determine whether miR-30a functions as a tumor suppressor gene via regulation of DBF4. Previous reports suggested that miR-30a may inhibit the proliferation and invasion of GC [22]. In agreement with these previous findings, MGC-803 and AGS gastric cell lines exhibited reduced proliferation after transfection with miR-30a mimics (Fig. 6B, C). qRT-PCR analysis showed that DBF4 expression was suppressed in the miR-30a mimic group and upregulated in the DBF4 overexpression group (Fig. 6A). CCK-8 and colony formation assays showed that the suppressive effect of miR-30a overexpression on cell proliferation was abrogated by overexpression of DBF4 (Fig. 6B, C). In order to further determine the effect of miR-30a-DBF4 axis on cell migration, would healing assay and transwell assays were used, and the results indicated that suppressive effect of miR-30a overexpression on cell migration was abrogated following DBF4 overexpression. (Figure 6D and E). Next, miR-30a alone or along with PEX-DBF4 was introduced into the two GC cells with 5-Fu treatment. CCK-8 analysis displayed that miR-30a mimics led to the decrease of the IC50 of 5-Fu, and DBF4 overexpression could reverse the miR-30 -overexpression effect on the IC50 of 5-Fu in MGC-803 and AGS cells (Fig. 6F). Taken together, these results suggested that DBF4 restored the effects of miR-30a on proliferation, migration and the 5-Fu-sensitivity of AGS and MGC-803 cells.

### Lactate in the tumor microenvironment induces aberrant expression of miR-30a and DBF4

**Fig. 7:**
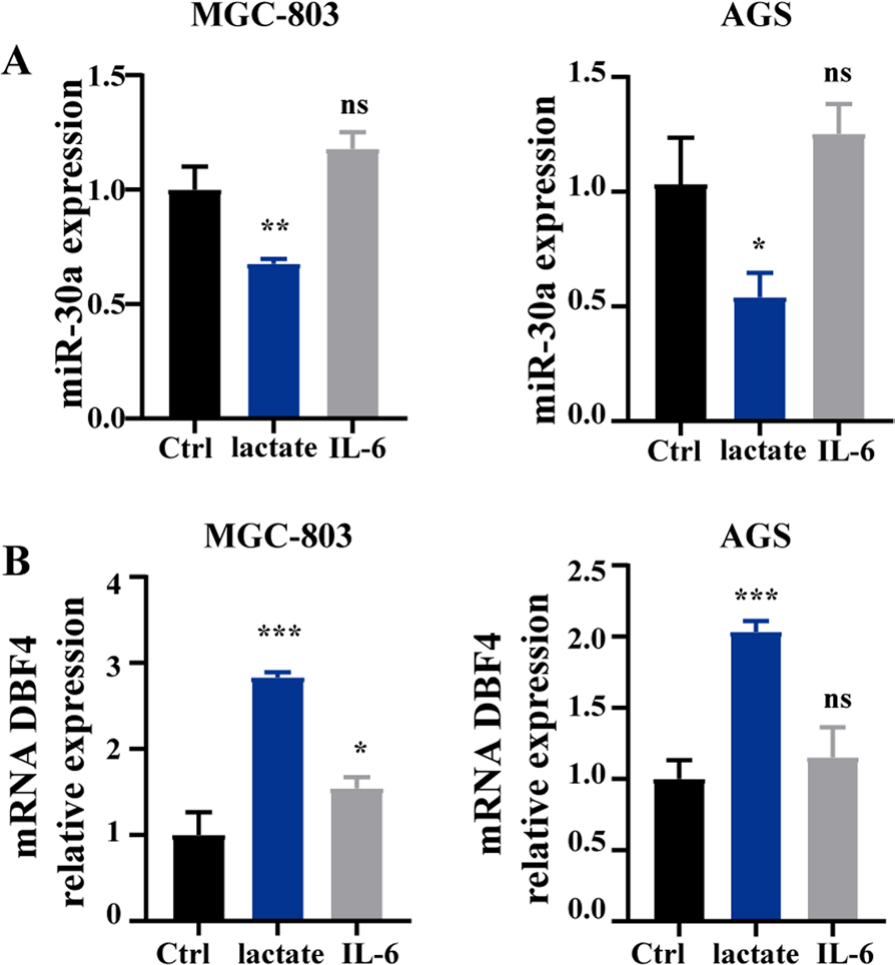
Lactate inhibits the expression of miR-30a and upregulates DBF4 in GC cells. MGC-803 and AGS cells were stimulated with lactate (50 mM) and IL-6 (20 ng/mL) for 24 h. **A** The expression of miR-30a in MGC-803 cells (left) and AGC cells (right) was detected by qRT-PCR. **B** The qRT-PCR was used to detect DBF4 mRNA expression in MGC-803 cells (left) and AGC cells (right). Data are presented as the mean ± SD (n = 3). ^*^P < 0.05, ^**^P < 0.01

In addition to dysregulated cell proliferation, tumors exhibit another dimension of complexity in that they contain a repertoire of recruited immune cells, metabolites such as lactate, and inflammatory factors such as interleukin-6 (IL-6). To determine the primary factor that induced the aberrant expression of miR-30a and DBF4, we stimulated GC cells with lactate and IL-6 in MGC-803 and AGS cells. As shown in Fig. 7A, lactate suppressed miR-30a expression and IL-6 showed a weak effect on miR-30a expression. By qRT-PCR analysis, we determined that lactate was the main factor inducing the aberrant expression of DBF4 (Fig. 7B). Together, our findings demonstrated that accumulation of lactate in the tumor microenvironment inhibited miR-30a expression and increased DBF4 expression.

## Discussion

Although gastric cancer (GC) is one of the most aggressive malignancies worldwide, there are limited treatment options available and the disease has a poor prognosis after local progression and metastasis [23]. Arguably one of the most fundamental traits of cancer cells is their ability to undergo continued proliferation, but little is known about the precise that regulate this process. Using the TCGA database and GSEA analysis, we identified the cell cycle pathway as one of the most enriched pathways in GC tissues. We also found that DBF4 had significantly higher expression in GC compared to normal tissues. Two GC cell lines, MGC-803 and AGS, were used in this study. CCK-8, colony formation, and western blotting assays were used to analyze GC cell proliferation, would healing and transwell assay were performed to detect GC cells migration, following transfection with DBF4 siRNA or a DBF4 overexpression vector. Cell proliferation assay were applied to confirm that DBF4 weakens the sensitivity of GC cells to 5-Fu. Furthermore, miR-30a, one of the most significantly downregulated miRNAs in GC tissue, was predicted and confirmed to be negatively correlated with DBF4 expression. qRT-PCR and western blots verified that miR-30a inhibited DBF4 expression, and a dual-luciferase reporter assay demonstrated that miR-30a directly inhibited the 3ʹ-UTR region of DBF4. Resulting from the Weinberg effect of tumor cells, a large amount of lactate accumulates in the tumor microenvironment. qRT-PCR experiments verified that lactate inhibited the expression of miR-30a and significantly increased the expression of DBF4. Ultimately, using TCGA databases for GC, we identified DBF4 as an important regulator of proliferation and migration in GC cells. Moreover, through prediction and experimental verification, we demonstrated the important role of the lactate-miR-30a-DBF4 axis in the development of GC, providing new targets and important theoretical guidance for the treatment of late-stage GC.

CDC7-DBF4 is a conserved serine/threonine kinase that plays an important role in initiation of DNA replication and DNA damage tolerance. DBF4 is the regulatory subunit, which is necessary for kinase activity and for targeting of various substrates [24]. In the last decade, the CDC7-DBF4 complex has emerged as a potential novel chemotherapeutic target. CDC7 kinase deficiency can induce significant apoptosis of tumor cells, while normal cells undergo a reversible cell cycle arrest [25–27]. However, the effect of DBF4 on GC proliferation and migration and the precise mechanisms involved remained unclear. To our knowledge, our study is the first to report that increased expression of DBF4 potentiated GC cell proliferation, as demonstrated by the CCK-8, colony formation, and western blot assays in two GC cell lines transfected with si-DBF4 or DBF4 overexpression vectors. 5-Fu is considered as one of the first‐line chemotherapy drugs in advanced gastric cancer(GC). And we also figured out that DBF4 weakens the sensitivity of GC cells to 5-Fu, which suggested that DBF4 may become a novel therapeutic targets in GC treatment. Furthermore, our results suggested that DBF4 also play an important role in the regulation of cancer cells migration.

The miRNAs are small non-coding RNAs that post-transcriptionally regulate the expression of many genes to control physiological and pathological processes, including the occurrence, growth, and progression of cancer [28]. Until now, approximately 200 different miRNAs have been implicated in the occurrence and treatment of GC, and numerous studies have suggested that miRNAs may play an important role in various types of cancer, including GC [28–30]. Deregulation of miR-30a has been shown to play a role in many human cancers, and significant downregulation of miR-30a has been detected in anaplastic thyroid carcinomas and non‑small cell lung cancer [31, 32]. In contrast, upregulation of miR-30a has been reported in glioma [33], which suggests a context-specific role for miR-30a across different cancer types. In the present study, miR-30a significantly inhibited the proliferation of GC cells, which is in agreement with the previous reports [22]. Furthermore, we demonstrated miR-30a regulated GC cells by inhibiting the expression of DBF4. Dual-luciferase reporter assays showed that miR-30a significantly suppressed the 3ʹ-UTR of DBF4. However, additional studies are required to determine the precise mechanism of this regulation. miR-30a was recently observed to be downregulated in GC, resulting from high promoter methylation induced by DNA methyltransferase [34]. In our study, we found that accumulation of lactate in the tumor microenvironment reduced the expression of miR-30a, suggesting that miR-30a may also be regulated by lactylation. Additional future experiments are needed to confirm this finding.

Our study is the first time to identify DBF4 as a critical tumor suppressor in GC. Upregulation of DBF4 increased proliferation of GC cells. Mechanistically, miR-30 suppressed the 3ʹ-UTR of DBF4 and inhibited its expression. Lactate in the tumor microenvironment was the primary factor that induced aberrant expression of miR-30a and DBF4. In conclusion, the lactate-miR-30a-DBF4 axis was a critical regulator of GC cell proliferation, migration, sensitivity to 5-Fu and may have potential to serve as a target for the treatment of gastric cancer.

## Data Availability

We hereby undertake that all data and materials are available.
